# Seronegative Arthritis and Whipple Disease: Risk of Misdiagnosis in the Era of Biologic Agents

**DOI:** 10.1155/2019/3410468

**Published:** 2019-10-13

**Authors:** Luca Quartuccio, Ivan Giovannini, Stefano Pizzolitto, Maurizio Scarpa, Salvatore De Vita

**Affiliations:** ^1^Rheumatology Clinic, Department of Medical Area, Academic Hospital Santa Maria della Misericordia, University of Udine, Udine, Italy; ^2^Institute of Pathology, Academic Hospital Santa Maria della Misericordia, Udine, Italy; ^3^Rare Diseases Regional Coordinating Centre, Academic Hospital Santa Maria della Misericordia, Udine, Italy

## Abstract

We report 2 cases of Whipple disease (WD), previously diagnosed as seronegative polyarthritis and treated for several years with immunosuppressive agents, accordingly. Both cases had been treated over years with cDMARDs and bDMARDs. The first patient was a 48-year-old male, who developed a life-threatening disease characterized by fever, significant weight loss, and bloody diarrhoea, supported with RBC transfusions. The second patient was a 55-year-old man, presenting with arthritis, fever, serositis, lymphadenopathy, thoracic rash, and systemic inflammation; at the beginning he was diagnosed as adult onset Still's disease. He was treated with steroids and antitumour necrosis factor agents, but showed no improvement. Both patients were eventually treated with antimicrobial therapy for WD with dramatic improvement and no clinical relapse in 6 months. This paper reviews the literature on WD mimicking chronic inflammatory arthritis. WD may lead to chronic seronegative arthritis that might often be misrecognized. Importantly, patients treated with bDMARDs and glucocorticoids might develop a life-threatening disease. Therefore, WD should be suspected and excluded in patients showing resistance or frequent recurrence of chronic arthritis, if seronegative, under treatment with bDMARDs, especially in the presence of new, unexpected sign and/or symptoms.

## 1. Introduction

Whipple disease (WD) is a rare infection disorder, first described by George Whipple in 1907, caused by the rod-shaped actinomycete, *Tropheryma whipplei*, and characterized by diarrhoea, weight loss, and arthralgia. Human beings are the only known host of *T*. *whipplei* and the spread of this infectious disease might be related to human-to-human transmission [1].

*T*. *whipplei* is commonly infecting humans, while WD is rare. Cases are rare and disproportionately associated with occupational exposure to soil or animals [2] and may mimic more common conditions. According to a report regarding 142 patients affected by *T*. *whipplei* [3], the main symptom is arthralgia (88/113, 78%), which might explain the frequent misdiagnosis as chronic inflammatory arthritis, such as rheumatoid arthritis (56/113, 50%). Fifty percent of patients receive immunosuppressive treatments which are responsible for a more rapid clinical progression (43%). Endocarditis is the second most frequent manifestation of *T. whipplei* [4], followed by neurologic symptoms. Other localized infections such as adenopathy, uveitis, pulmonary involvement, or frank arthritis are sporadic, but still may cause misdiagnoses. Diagnosis of WD can be established by periodic acid-Schiff (PAS) staining of inclusion bodies within lamina propria macrophages in biopsies of the small intestine and by polymerase chain reaction (PCR) [1]. In the absence of duodenal histologic involvement, localized infections were defined by specific positive *T. whipplei* PCR results obtained using samples of other tissues and body fluids. Early diagnosis should enable appropriate treatment and improve the prognosis, and prolonged antibiotic treatment often leads to complete remission [1]. The disease could develop in decades because of a very long doubling time (up to 18 days) and only in predisposed patients [5]. Patients showing seronegative oligoarthritis or polyarthritis could be mistakenly treated with immunosuppressive agents, including biologic drugs, even for a long time. Here, we report two cases which we consider emblematic to include WD as mandatory in the differential diagnosis of seronegative arthritis.

## 2. Case Presentation

All procedures performed in our study involving human subjects were in accordance with the ethical standards of the institutional and/or national research committee and with the 1964 Helsinki Declaration and its later amendments or comparable ethical standards. Informed consent was obtained from the patients included in the study.

### 2.1. Patient 1

The first patient was a 48-year-old Caucasian male with progressive and unintended weight loss, daily diarrhoea (initially without bleeding), and fever.

In the past medical history, he had a previous diagnosis of seronegative spondyloarthritis because of 7-year intermittent joint swelling, early morning stiffness associated with inflammatory back pain and increased C-reactive protein (CRP), and a familiar history of psoriasis. No history of travel or surgery was recorded. Therapeutic trials with different antirheumatic drugs included methotrexate, hydroxychloroquine, etanercept (ETN), and finally, tocilizumab (TCZ).

The most recent medical history of the first patient was characterized by disease relapse with fever and systemic inflammation (CRP 20 mg/l) without any response to a medium dose of glucocorticoids in May 2017. Before May 2017, he was finally treated with monthly TCZ 8 mg/kg intravenously, which was effective on his clinical manifestations, allowing the resolution of arthritis, and fever and led to normalization of CRP within 3 months of therapy and, notably, lasting 2 years. In April 2017, abdominal pain, daily fever (around 38°C), and daily diarrhoea without bleeding started. The patient reported a relevant weight loss of 8 kg during the last 3 months.

Importantly, physical examination showed no evidence of an arthritis flare, while cutaneous lesions on the lower limbs that may resemble a cutaneous vasculitis or atypical psoriasis. Laboratory exams revealed CRP 72 mg/l, hemoglobin 10.7 g/l, HLA-B38 positive, and HLA-B27 and B51 negative. Rheumatoid factor (RF), anticitrullinated protein antibodies (ACPAs), ANA, and ANCA autoantibodies were negative. The day after the admission in our hospital ward, the patient presented bloody diarrhoea with a significant decrease in the hemoglobin level in 24 hours (from 10.7 g/dl to 8.4 g/dl). Thus, he urgently underwent colonoscopy and gastrointestinal arteriography, but without revealing the site of bleeding.

We tested also Vidal–Wright reaction, CMV, HBV, HCV, *H. pylori*, TB test, rotavirus, adenovirus, norovirus, Yersinia, Shigella, Salmonella, Campylobacter, Clostridium, and blood cultures, but all tests resulted negative.

Suspecting an intestinal vasculitis or a chronic inflammatory bowel disease, we administered methylprednisolone 1 g daily for three days with the regression of diarrhoea and fever. In order to find the site of bleeding, he underwent a gastrointestinal bleeding scintigraphy and CT-PET, which revealed a diffuse uptake in the small bowel.

**Figure 1:**
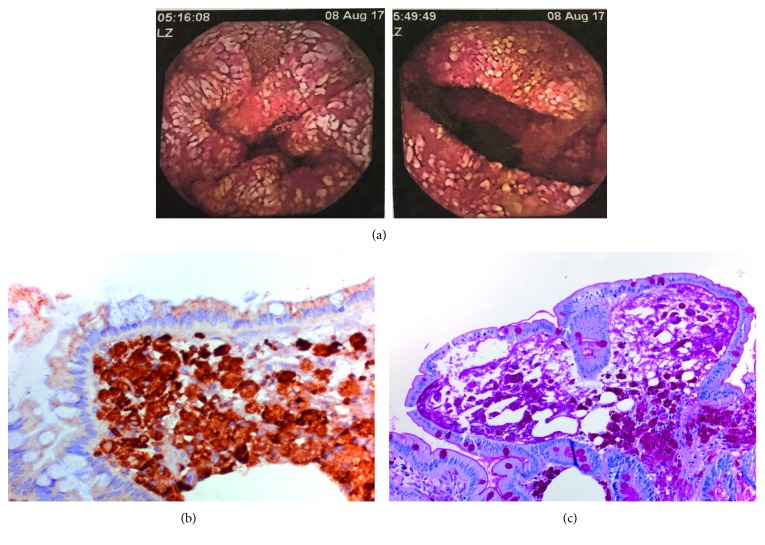
Macroscopic and microscopic descriptions of WD in case number 1. (a) Intestinal lipodystrophy in duodenum. (b) CD68 positive for macrophage-duodenal biopsy. (c) PAS-positive granules-duodenal biopsy.

Because of persistent weight loss, we introduced parenteral nutrition and explored other causes of malabsorption syndrome, such as WD; thus, the patient underwent gastroscopy. The histological findings resulted in PAS-positive granules in macrophages (intestinal lipodystrophy) (Figure 1). *T. whipplei* was then detected in blood, feces, and saliva samples by PCR in an international reference laboratory.

**Figure 2:**
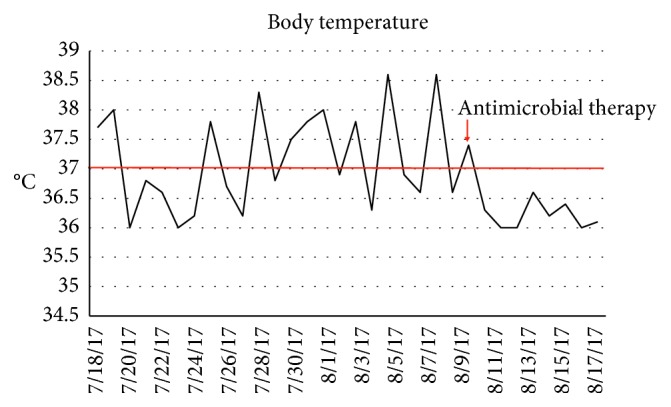
Course of body temperature (°C) in case number 1.

**Figure 3:**
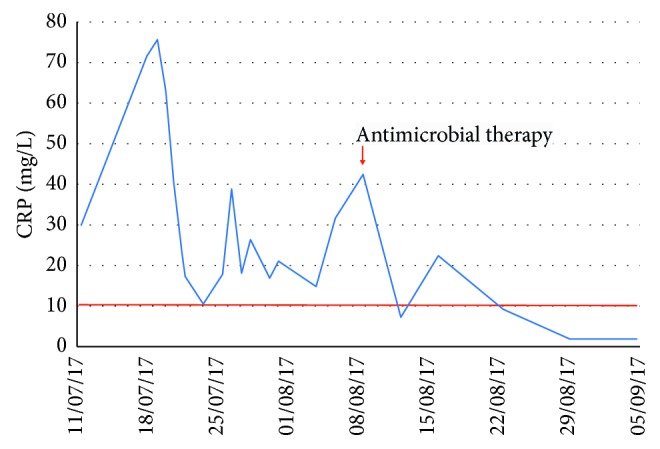
Course of CRP (mg/l) in case number 1.

Intravenous ceftriaxone (2 g daily for one week) was started followed by trimethoprim-sulfamethoxazole 160/800 mg 3 times a day with dramatic improvement. There were no relapses after 6 months and was a significant weight recovery (Figures 2 and 3).

### 2.2. Patient 2

The second patient was a 55-year-old Caucasian male, with a history of symmetric polyarthritis and systemic inflammation (CRP 130 mg/l) without fever, previously diagnosed as seronegative rheumatoid arthritis in 2012. No history of travel or surgery was recorded. Over the years, he was treated with cDMARDs (methotrexate) and bDMARDs (certolizumab, ETN, TCZ, abatacept, and adalimumab).

The disease relapsed with arthritis (synovitis demonstrated by US), fever (39°C), and thoracic discomfort, suspicious for pleurisy in September 2017. Previous treatments provided only a partial clinical benefit; however, the systemic inflammation with CRP ranging from 20 to 40 mg/l persisted. There was not any improvement with the introduction of colchicine. After switching to anakinra, we even registered a clinical worsening with fever, arthritis, laterocervical lymphadenopathy, and thoracic rash along with a higher systemic inflammation (CRP 213 mg/l).

Many infections were excluded (i.e., CMV, EBV, chlamydia, mycoplasma, TB, Salmonella, Shigella, and *Borrelia burgdorferi*) as well as autoinflammatory diseases (negative genetic tests for Mediterranean fever and no improvement with colchicine and anakinra treatment, as reported) and, initially, WD by negative duodenum histopathology (no signs of PAS-positive granules in the duodenum samples).

In the absence of other diagnosis, the adult onset Still's disease appeared the most probable one. The patient was then treated with steroid 0.5 mg/kg/day, with clinical improvement. Infliximab was started and steroid progressively tapered, but with recurrence of fever and systemic inflammation (CRP 68 mg/l).

**Figure 4:**
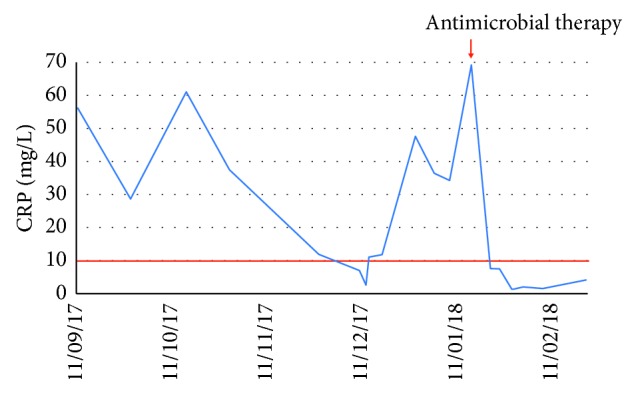
Course of CRP (mg/l) in case number 2.

Despite the PAS negativity in the duodenal biopsy, the patient was then treated with ceftriaxone, showing a dramatic and rapid clinical and laboratory improvement (Figure 4).

**Figure 5:**
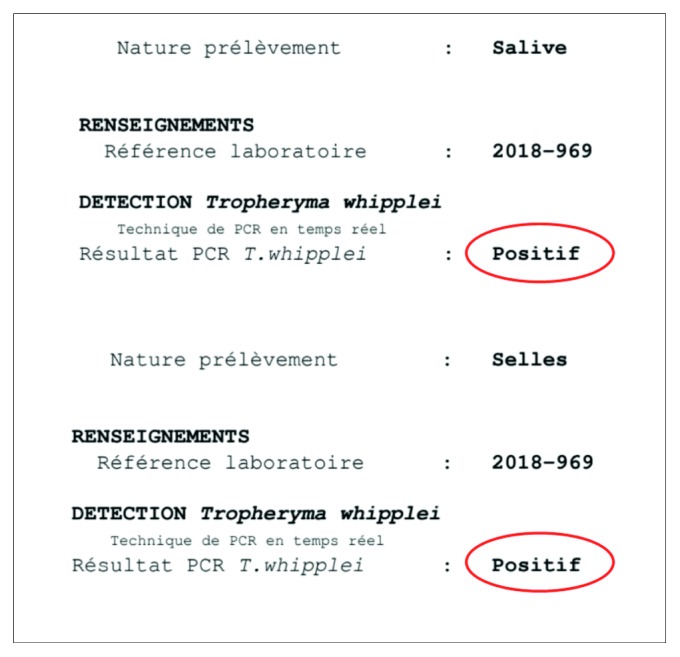
*Tropheryma whipplei* detection by PCR on saliva and stool in case number 2 (original report from the Reference Laboratory of Marseille, France).

**Figure 6:**
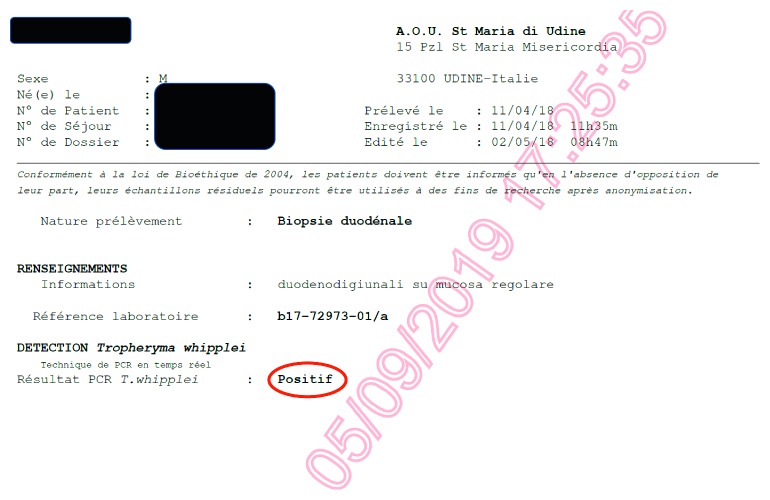
*Tropheryma whipplei* detection by PCR on duodenum sample in case number 2 (duplicate from the original report from the Reference Laboratory of Marseille, France).

Thus, the duodenal samples, as well as other samples from stool and saliva, collected before the introduction of the antibiotic therapy, were analyzed for *T. whipplei* by PCR in the Reference Center of Marseille, resulting all positive (Figures 5 and 6). Finally, the patient was treated with doxycycline 100 mg twice daily and hydroxychloroquine 6 mg/kg/day, with no relapse after 6 months.

## 3. Discussion

*T. whipplei* is a ubiquitous Gram-positive microorganism that is rarely associated with symptomatic disease, namely, WD [1]. *T. whipplei* is a commensal organism and not an obligate pathogen. Up to 7% of individuals are healthy carriers with positive stool PCR tests [6]. The systemic infection involves especially the digestive tract. The epidemiology of WD is limited by small sample size and case series design. The incidence of WD has been estimated at about 0.5 to 1/1000000 people [7]. In a recent large population-based study, the overall prevalence of WD in the USA was 9.8 cases per 1 million people. It equally affects men and women and is more common in Caucasians and middle-aged people [8]. The way of transmission may involve passage of the bacterial agent from the environment into the body through the gastrointestinal tract, followed by fecal-oral transmission. However, person-to-person transmission through the oral-oral route cannot be ruled out [9]. Farmers and individuals exposed to soil and animals may be at higher risk. Besides the classical presentations with chronic diarrhoea and malabsorption, clinical presentations include endocarditis, central nervous system (CNS) involvement, uveitis, arthritis, and diskitis without gastrointestinal manifestations and/or with normal gastrointestinal histology [9]. Indeed, patients with WD were more likely to have associated arthritis, CNS manifestations, endocarditis, diabetes, malignancy, dementia, vitamin D deficiency, iron deficiency, chemotherapy, weight loss, abdominal pain, and lymphadenopathy [10].

WD often leads to a chronic seronegative arthritis that could be often misdiagnosed [11, 12]. Interestingly, 1.58% of patients with unexplained arthritis and no other evidence of WD were positive for *T. whipplei* [13]. Articular involvement often characterizes the onset of WD. The mean time from the development of articular symptoms to the diagnosis is almost 7 years [8], but there are some reports focusing the attention on the very delay of the diagnosis of WD, in the absence of gastrointestinal symptoms [14, 15]. Arthritis has been reported in 41%–61% of cases [8]. In a retrospective single-center cohort study of 7 patients, all patients presented with polyarthritis with a predominantly symmetric pattern, with erosion in 3 out of 7 patients. The most affected joints were wrists, metacarpophalangeal joints, and knees, followed by proximal interphalangeal joints, hips, elbow, and shoulder. All patients had increased CRP, while RF and ACPA were absent, and all were initially misclassified as affected by seronegative RA. Six patients received DMARD treatment consisting of methotrexate and/or leflunomide, and three were also treated with at least one bDMARD. Most patients showed an inadequate response. In all patients, *T. whipplei* was detected in synovial fluid by PCR. Notably, gastrointestinal symptoms and other extra-articular manifestations were absent, mild, or nonspecific. All patients had good treatment responses with improvement of arthritis and extra-articular manifestations upon started antibiotic therapy [12]. Even if gastrointestinal involvement is usually demonstrated by histological or PCR tests, a few patients, however, have chronic focal joint infection with normal gastrointestinal biopsy specimens, even by PCR [16]. Thus, unexplained intermittent oligoarthritis or polyarthritis, especially of the large joints, in middle-aged men should suggest excluding WD, even in the absence of gastrointestinal symptoms [17]. Chronic polyarthritis in a symmetric distribution is less common, and the small joints are usually spared. Rarely, patients with prolonged untreated WD may develop erosions, and the progression to the late stage of ankylosis may occur only after many years [8]. Notably, patients treated with TNF-alpha inhibitors and glucocorticoids could reveal a life-threatening disease, triggering visceral disorders [18–21]. On the contrary, the present case reports showing a clear, even if transient, clinical response for a long time under immunosuppressive agents, demonstrate that the course of arthritis does not really allow the clinician to definitely rule out such a chronic infection. Notably, clinicians should be aware that especially TCZ as a potent inhibitor of IL-6 is able to abolish systemic features, such as fever or CRP elevation, along with arthritis, while the infection spreads slowly, as clearly reported in our first patient.

A recent literature search on 19 studies reported the use of immunosuppressive drugs, particularly therapy with TNF inhibitors, before the diagnosis in 41 patients with WD [22]. As arthritis may precede the diagnosis of WD by many years, a relevant percentage (up to 50% in some reports) of patients is treated with immunomodulatory drugs or with biologics. Indeed, complicated WD course or *T. whipplei* endocarditis following medical immunosuppression, particularly after TNF inhibitors, have been reported [23]. The histological detection of macrophage-containing PAS-positive granules in the duodenum lamina propria is considered the standard diagnostic method [1]. Notably, as in our second case, negative PAS particles do not completely rule out WD. Another diagnostic procedure, critical in immunosuppressed patients, is the *T. whipplei* PCR, especially in strong clinical suspicion [13, 16]. This test can be made on various samples, from saliva to stools and also on synovial fluid, but the highest specificity relies on the duodenal biopsy [6], even if negative small bowel PAS and PCR do not definitely exclude the diagnosis of WD, and blood PCR alone is insensitive for active infection [24]. Importantly, in the case of seronegative arthritis (especially in the absence of systemic symptoms), synovial fluid should always be collected, if possible, and then analyzed also by T*. whipplei* PCR, based on clinical suspicion, since in Whipple arthritis, the diagnosis is made by PCR analysis of the synovial fluid. The gastrointestinal analyses (PAS staining, histology, and PCR) are done in order to exclude additional systemic or intestinal manifestations [25, 26]. Doxycycline together with hydroxychloroquine is the first-line choice for WD without neurological involvement (defined as absence of clinical neurological abnormalities and a negative PCR assay on the cerebrospinal fluid). In the past, the most widely recommended regimen was oral trimethoprim 160 mg and sulfamethoxazole 800 mg twice daily for 1 to 2 years. In any case, treatment generally includes primary therapy for 2 weeks with intravenous antibiotics capable of reaching high levels in the cerebrospinal fluid, such as ceftriaxone. Association with glucocorticoids has been suggested to avoid the immune reconstitution syndrome, which affects 2%–10% of patients [27]. Antibiotic therapy usually promptly provides improvement in the clinical and laboratory abnormalities [28], but it is of great importance to continue the treatment for at least 2 years or even preferably throughout life [29].

## 4. Conclusions

WD should be suspected and definitely ruled out in patients affected by seronegative arthritis, who show a disease course characterized by resistance or recurrence of the disease, along with the development of new, unexpected symptoms or signs (in particular fever, serositis, and/or gastrointestinal symptoms). Duodenal biopsy is the standard diagnostic procedure; however, in those patients with negative result on duodenal biopsy, but high level of suspicion and risk of complication due to immunosuppression, *T*. *whipplei* PCR should be performed on all the available body tissues or fluids, in particular the synovial fluid. Finally, a correct disease classification of patients remains an essential step for an appropriate use of targeting treatments in systemic rheumatic diseases, especially in seronegative arthritis since selective inhibitors of proinflammatory cytokines may masquerade symptoms and signs of chronic systemic infections.

## Additional Points

(1) Whipple disease should be considered in the differential diagnosis of seronegative arthritis. (2) All the available body tissues or fluids should be investigated by *T. whipplei* PCR. (3) Biologic agents can mask symptoms and signs of such a chronic infection, in particular IL-6 inhibitors. (4) Correct classification is an essential step for using biologics properly. (5) Systemic symptoms along with arthritis should alert the clinician to suspect Whipple disease.
